# Reduced number of axonal mitochondria and tau hypophosphorylation in mouse P301L tau knockin neurons

**DOI:** 10.1016/j.nbd.2015.10.007

**Published:** 2016-01

**Authors:** Teresa Rodríguez-Martín, Amy M. Pooler, Dawn H.W. Lau, Gábor M. Mórotz, Kurt J. De Vos, Jonathan Gilley, Michael P. Coleman, Diane P. Hanger

**Affiliations:** a King's College London, Institute of Psychiatry, Psychology & Neuroscience, Maurice Wohl Clinical Neuroscience Institute, Department of Basic and Clinical Neuroscience, London, SE5 9NU, UK; b Signalling Programme, The Babraham Institute, Cambridge CB22 3AT, UK

**Keywords:** DIV, days in vitro, E18, embryonic day 18, EGFP, enhanced green fluorescent protein, GSK-3, glycogen synthase kinase-3, PBS, phosphate-buffered saline, PP, protein phosphatase, SDS, sodium dodecyl sulphate, Dementia, Tau, Mitochondria, Axonal transport, Phosphorylation, Membrane

## Abstract

Expression of the frontotemporal dementia-related tau mutation, P301L, at physiological levels in adult mouse brain (KI-P301L mice) results in overt hypophosphorylation of tau and age-dependent alterations in axonal mitochondrial transport in peripheral nerves. To determine the effects of P301L tau expression in the central nervous system, we examined the kinetics of mitochondrial axonal transport and tau phosphorylation in primary cortical neurons from P301L knock-in (KI-P301L) mice. We observed a significant 50% reduction in the number of mitochondria in the axons of cortical neurons cultured from KI-P301L mice compared to wild-type neurons. Expression of murine P301L tau did not change the speed, direction of travel or likelihood of movement of mitochondria. Notably, the angle that defines the orientation of the mitochondria in the axon, and the volume of individual moving mitochondria, were significantly increased in neurons expressing P301L tau. We found that murine tau phosphorylation in KI-P301L mouse neurons was diminished and the ability of P301L tau to bind to microtubules was also reduced compared to tau in wild-type neurons. The P301L mutation did not influence the ability of murine tau to associate with membranes in cortical neurons or in adult mouse brain. We conclude that P301L tau is associated with mitochondrial changes and causes an early reduction in murine tau phosphorylation in neurons coupled with impaired microtubule binding of tau. These results support the association of mutant tau with detrimental effects on mitochondria and will be of significance for the pathogenesis of tauopathies.

## Highlights

• Expression of P301L tau reduces the number of axonal mitochondria in mice.
• Motile mitochondria exhibit increased volume in axons of neurons with P301L tau.
• P301L tau expressed in knockin mice is hypophosphorylated.
• The P301L tau mutation impairs microtubule binding but does not affect tau membrane localization.

## Introduction

The microtubule-associated protein tau is expressed mainly in neurons where it stabilises microtubules and is involved in neurite outgrowth (Hernandez et al., 2002, Lee and Rook, 1992). The microtubule-binding domain of tau is located in its carboxy-terminal half and tubulin binding is regulated by tau phosphorylation state (Bramblett et al., 1993, Busciglio et al., 1995). The amino-terminal projection domain of tau interacts with components of the plasma membrane and is also affected by tau phosphorylation state (Bramblett et al., 1993, Busciglio et al., 1995, Pooler et al., 2012).

The human *MAPT* gene is located on chromosome 17 and comprises 16 exons. Exclusion or inclusion of exon 10 gives rise to tau isoforms with three (3R) or four (4R) microtubule binding repeats (Andreadis et al., 1992, Goedert et al., 1989). In the developing brain, 3R tau isoforms predominate, whereas in adult human brain 3R and 4R tau are expressed in approximately equal amounts. Mutations in *MAPT* cause frontotemporal dementia with parkinsonism linked to tau mutations on chromosome 17 (FTDP-17T) (Hutton et al., 1998, Poorkaj et al., 1998, Spillantini et al., 1998), characterised by intraneuronal aggregates of insoluble, highly phosphorylated tau. FTDP-17T and other neurodegenerative diseases with CNS tau aggregates are collectively referred as tauopathies (Ballatore et al., 2007, Gallo et al., 2007). Disease-associated mutations in *MAPT* occur as exonic missense mutations (e.g. P301L), silent mutations (e.g. N279N), or intronic mutations that affect exon 10 splicing regulatory elements and thereby alter the 4R/3R tau isoform ratio (D'Souza et al., 1999, Grover et al., 1999, Spillantini et al., 1998). However, not all of the known mutations in *MAPT* result in altered tau splicing and furthermore, the molecular mechanisms that link these mutations to the observed pathological and clinical features of the tauopathies are not well understood.

Many transgenic mouse lines that model tauopathies have been generated by overexpression of either wild-type or FTDP-17T mutant tau (reviewed in Denk and Wade-Martins, 2009, Noble et al., 2010). Axonal degeneration and transport impairments have been described in several of these mouse models, with more frequent mature filamentous tau pathology occurring in mice overexpressing mutant tau. However, differences in the expression of exogenous tau due to the use of heterologous promoters, and an imbalance in tau isoform expression by overexpression of individual isoforms of human tau, are significant limitations in many of these models. For example, P301L or P301S tau expressed under the control of different promoters including prion (Lewis et al., 2000), Thy 1 (Allen et al., 2002, Terwel et al., 2005) and calcium-calmodulin kinase II (Santacruz et al., 2005), each result in different tau expression patterns and variable phenotypic outcomes.

We created a transgenic tau knock-in (KI) mouse expressing physiological levels of murine tau and harbouring mutant P290L tau, equivalent to human P301L tau (Gilley et al., 2012). We used this mouse line to investigate the impact of P301L tau on FTDP-17T-associated tau pathology and neural dysfunction (Gilley et al., 2012). Overt tau pathology was not observed and interestingly, we found that the overall level of tau phosphorylation was reduced in adult KI-P301L mice (Gilley et al., 2012). However, these transgenic mice exhibited age-dependent changes in mitochondrial axonal transport.

Mitochondria are highly dynamic organelles that undergo continuous bi-directional movements, combined with frequent fission and fusion events (Schulz et al., 2012). Dysregulation of mitochondrial activity and transport is associated with a number of age-related neurodegenerative disorders (De Vos et al., 2008, Exner et al., 2012, Lin and Beal, 2006). Recent findings also implicate defective mitochondrial function and dynamics induced by amyloid beta-peptide and/or tau in the pathogenesis of Alzheimer's disease (Amadoro et al., 2014, Eckert et al., 2013, Manczak and Reddy, 2012).

To gain insight into the mechanisms underlying the axonal transport defect observed in KI-P301L mice, we characterised the effects of tau on axonal mitochondrial transport in primary cortical neurons and investigated tau phosphorylation. We found that the total number of mitochondria in axons was reduced and the volume of individual motile mitochondria was significantly increased in neurons derived from KI-P301L mice. We also found that tau hypophosphorylation observed in adult mouse brain was recapitulated in cultured cortical neurons. Our results suggest that physiological expression of mutant P301L tau decreases tau phosphorylation and reduces the number of axonal mitochondria at a very early stage of development, supporting the association of mutant tau with dysregulation of mitochondrial activity.

## Material and methods

### Mouse maintenance and generation

All mice were bred and housed in accordance with the UK Home Office Animals (Scientific Procedures) Act, 1986. KI-P301L mice were generated in a C57BL/6 background by (Gilley et al., 2012). Briefly, the human FTDP-17 P301L mutation was targeted to the homologous codon in exon 10 of the mouse *Mapt* gene — P290 in 2N4R murine tau. Correct targeting of the knockin allele was verified by Southern blotting and mice were genotyped by Southern blotting or PCR. Expression and normal splicing of *Mapt* messenger RNA derived from the “P301L” tau knockin allele was confirmed by RT-PCR (Gilley et al., 2012).

### Cortical neuronal culture and transfection

Embryonic day 18 (E18) wild-type and P301L tau-expressing mouse cortical neurons were cultured as described previously (Cuchillo-Ibanez et al., 2008). Cortical neurons were transfected using either calcium phosphate (Promega, Madison, USA) or Lipofectamine 2000 (Invitrogen, Carlsbad, CA, USA) following the manufacturers' instructions.

### Time-lapse imaging and analysis of mitochondria in transfected neurons

E18 rat cortical neurons 7 DIV were co-transfected with plasmids expressing enhanced green fluorescent protein (EGFP) or DsRed-Mito (Invitrogen, Carlsbad, CA, USA) and time-lapse images were recorded after 24–48 h. Coverslips were placed in a sealed chamber and the cells were maintained at 37 °C using an objective heater (Tempcontrol 37–2, Zeiss, Jena, Germany) and “The Box” Microscope Temperature Control System (Life Imaging Services Basel, Switzerland) on the stage of an Axiovert 200 M Zeiss microscope equipped with a Lambda LS Xenon-Arc light source (Sutter Instrument Company, Novato, CA, USA). Images of mitochondrial movement were collected at 3 s intervals for 10 min using a Plan-ApoChromat 40 × 1.4 NA oil immersion objective, an EGFP/DsRed filter set (Chroma Technology Corp., Rockingham, VT, USA) and an AxioCam MRm camera. Image analysis was performed with ImageJ developed by Wayne Rasband (NIH, Bethesda, MD, USA; http://rsb.info.nih.gov/ij/). EGFP expression enabled identification of transfected neurons and tracing of axons from the neuronal cell body to the growth cone, as we reported previously (Morotz et al., 2012). Mitochondrial kinetic parameters were assessed using Difference Tracker, a programme consisting of 2 plugins for the ImageJ software (Andrews et al., 2010). The contrast and despeckle functions were applied to all images. The difference filter plugin was used with minimum difference 10 and difference frame onset 4, and for the mass particle tracker plugin the settings for the initial flexibility and subsequent flexibility were 25 and 20, respectively. Quantitation and statistical analyses (Student t-test) were performed using Excel and Prism software (GraphPad Software Inc., La Jolla, CA, USA). Image analysis for parameters (angle, average size, circularity, perimeter, solidity, primary axis and secondary axis) of all mitochondria (stationary and motile) was performed using ImageJ.

### Gel electrophoresis and Western blots

10^6^ neurons per well of a 6-well plate were rinsed with phosphate-buffered saline (PBS) at 4 °C and cells were scraped into hot (2 ×) Laemmli sample buffer. Proteins were separated on 10% (*w*/*v*) sodium dodecyl sulphate (SDS) polyacrylamide gels and transferred to nitrocellulose. Membranes were probed with antibodies to total tau (rabbit polyclonal, DAKO, Glostrup, Denmark) and monoclonal Tau5 (Sigma-Aldrich, Gillingham, Dorset, UK), phosphorylated tau (polyclonal pT231 and pS404, Cell Signalling, Danvers, MA, USA and PHF1, a kind gift from Professor Peter Davies, Albert Einstein College of Medicine, New York), dephosphorylated tau (monoclonal Tau1, Millipore, Billerica, MA, USA), PP1 and PP2 (Santa Cruz, Dallas, Texas, USA), Opa1 (BD Transduction Laboratories), Mfn2 and monoclonal β-actin (both from Sigma, Gillingham, Dorset, UK). Antigens were visualised using an Odyssey scanner (Li-Cor Biosciences, Lincoln, Nebraska, USA).

### Microtubule binding assay

Assays for microtubule binding of tau were performed as described previously (Rodriguez-Martin et al., 2013). Wild-type and KI-P301L-expressing 12 DIV cortical neurons were rinsed in warm PBS, and suspended in warm microtubule-stabilising buffer (80 mM PIPES/KOH pH 6.8, 1 mM GTP, 1 mM MgCl_2_, 1 mM ethylene glycol-bis(2-aminoethyl)-N,N,N′,N′-tetraacetic acid (EGTA), 0.5% (*w*/*v*) Triton X-100, 30% (*v*/*v*) glycerol), containing 1 mM phenylmethylsulfonylfluoride, Complete protease inhibitor (Roche, Basel, Switzerland), 0.5 μM okadaic acid (Calbiochem Billerica, MA, USA) and 10 μM taxol (Sigma-Aldrich, Gillingham, Dorset, UK). Cell suspensions were centrifuged at 5000 *g* for 10 min at ambient temperature and an aliquot of the supernatant was retained as the post-nuclear lysate (input). The remaining post-nuclear lysate was centrifuged at 100,000 *g* for 1 h at ambient temperature. The supernatant, containing unbound tau, was retained and the pellet, containing microtubule-bound tau, was rinsed twice, and resuspended in microtubule-stabilising buffer. Proteins in each fraction were separated on 10% (*w*/*v*) SDS polyacrylamide gels and blots were probed with tau polyclonal antibody (DAKO, Glostrup, Denmark), as above.

### Dephosphorylation of neuronal lysates

Lambda phosphatase (New England Biolabs, Ipswich, MA, US) was used to dephosphorylate neuronal cell lysates as described (Hanger et al., 2002).

### Cell fractionation

Mouse cortical neurons cultured on 6-well plates were lysed in hypotonic buffer (10 mM sodium bicarbonate containing 20 mg/mL deoxyribonuclease I (DNase I), 1 mM sodium orthovanadate and complete protease inhibitor cocktail (Roche, Mannheim, Germany)), disrupted by sonication (five strokes with probe sonicator) on ice and processed as previously described (Pooler et al., 2013). Briefly, cell lysates were centrifuged at 720 *g* for 5 min at 4 °C, and the resultant supernatant was centrifuged at 100,000 *g* for 1 h at 4 °C. The final supernatant (cytosolic fraction) and the pellet (membrane fraction) were resuspended in Laemmli sample buffer, and analysed on Western blots probed with total tau polyclonal antibody (DAKO) and dephosphorylated tau antibody Tau1, as above.

### RT-PCR

Total RNA was isolated from primary cortical neurons using TRIzol reagent (Invitrogen, Carlsbad, CA, USA). Reverse transcription was performed using the Multiscribe RT kit (Applied Biosystems, Carlsbad, CA, USA) with random oligo(dT). The reverse transcription conditions were 10 min at 25 °C, 30 min at 48 °C, and a final step of 5 min at 95 °C. Reverse-transcribed RNA was amplified by PCR under the following conditions: 95 °C for 2 min, 30 cycles of 30 s at 94 °C, 30 s at 65 °C, 20 s at 72 °C and a final extension step for 10 min at 72 °C. The sequences of the primers used were: forward primer (exon 9), 5′-CTGAAGCACCAGCCAGGAGG-3′; reverse primer (exon 13), 5′-TGGTCTGTCTTGGCTTTGGC-3′. The predicted sizes for the corresponding PCR products were 274 base pairs for 3R tau and 367 base pairs for 4R tau. RT-PCR products were separated by electrophoresis in 1.5% (*w*/*v*) agarose gels and stained with ethidium bromide.

## Results

### Reduction of axonal mitochondrial number in P301L tau neurons

**Fig. 1:**
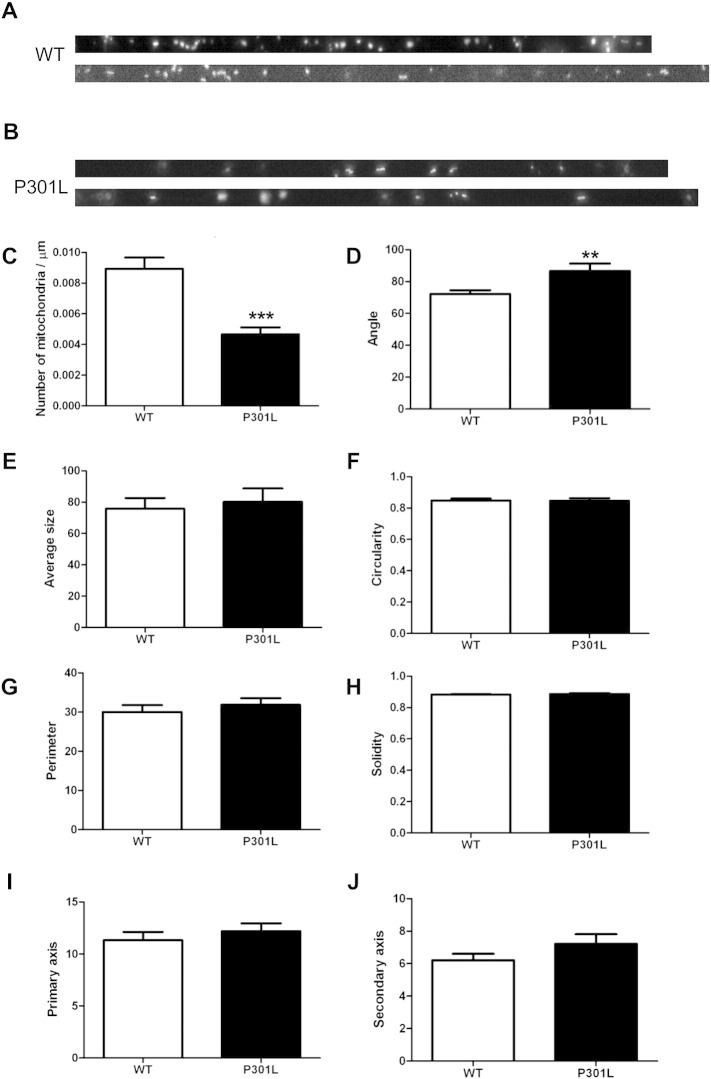
P301L tau reduces axonal mitochondria number and increases mitochondrial orientation angle in axons. Representative images of (A) wild-type (WT) and (B) knock-in P301L (P301L) straightened time-lapse movie. (C) Quantification of the number of mitochondria per μm axon. (D–J) Detailed analyses of time-lapse recordings: angle (D), average size (E), circularity (F), perimeter (G), solidity (H), primary axis (I) and secondary axis (J) of all mitochondria (stationary and motile) are shown as mean ± SEM (n = 28 for WT, n = 15 for P301L, Student t-test, P < 0.01**, P < 0.001***).

In a previous in vivo study, we found that adult KI-P301L tau mice exhibit age-dependent differences in axonal mitochondrial transport in isolated tibial nerve (Gilley et al., 2012). Whilst retrograde movement of mitochondria was unchanged, anterograde mitochondrial flux was increased in younger KI-P301L tau mice and decreased in older mice, relative to their wild-type counterparts. To investigate the age-dependency of these effects, we determined the effect of the P301L tau mutation on mitochondria in cultured neurons. Mouse cortical neurons 7 DIV (days in vitro) from wild-type and KI-P301L tau mice were co-transfected with plasmids expressing a mitochondrial targeting sequence fused to DsRed (DsRed-Mito), to label mitochondria, and EGFP, to visualise cell bodies and axons. Neurons expressing low levels of the exogenous proteins were selected to avoid potential artefacts due to over-expression. Movement of mitochondria was recorded at 3 s intervals over a period of 10 min using time-lapse microscopy at 24–48 h after transfection (Cuchillo-Ibanez et al., 2008, Morotz et al., 2012). Time-lapse movies were transformed into kymographs (wild-type, n = 28, P301L tau, n = 15) and the number of mitochondria present in the portion of axons recorded was calculated (Fig. 1A, B). Quantitation of the total mitochondria in axons revealed a 50% reduction in the number of mitochondria per μm in axons of neurons expressing P301L tau compared with wild-type tau (Fig. 1C). Interestingly, we found that the angle that defines the orientation of mitochondria in the axon was increased in neurons expressing P301L tau (Fig. 1D). In contrast, there were no differences between the average size, circularity, perimeter, solidity, primary axis or secondary axis of mitochondria in neurons expressing wild-type and P301L tau (Fig. 1E–J).

The number of stationary and motile mitochondria moving in both anterograde and retrograde directions was determined in wild-type and P301L tau-expressing neurons and expressed as a proportion of the total number of mitochondria (Table 1). This analysis revealed that approximately 20% of mitochondria are motile and 80% are stationary in neurons expressing either wild-type or mutant P301L tau. Of the moving mitochondria, 58% and 57% travelled anterogradely, and 42% and 43% travelled retrogradely, with respect to wild-type and P301L tau. Therefore, the presence of P301L tau did not influence the relative proportions of motile and stationary mitochondria or their direction of axonal transport.

**Fig. S1:**
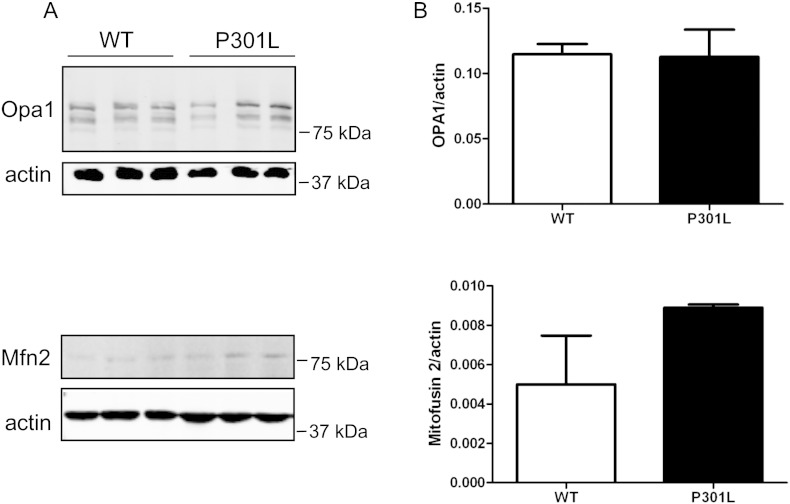
Levels of mitochondrial metabolism markers Opa1 and Mfn2. (A) Lysates of wild-type (WT) and P301L 8 DIV neurons were analysed on western blots using antibodies against optic atrophy 1 (Opa1), mitofusin 2 (Mfn2), and β-actin. (B) Quantification of Opa1 or Mfn2, relative to β-actin. No differences were found between wild-type and P301L tau neurons (mean ± S.E.M., n = 3).

**Fig. 2:**
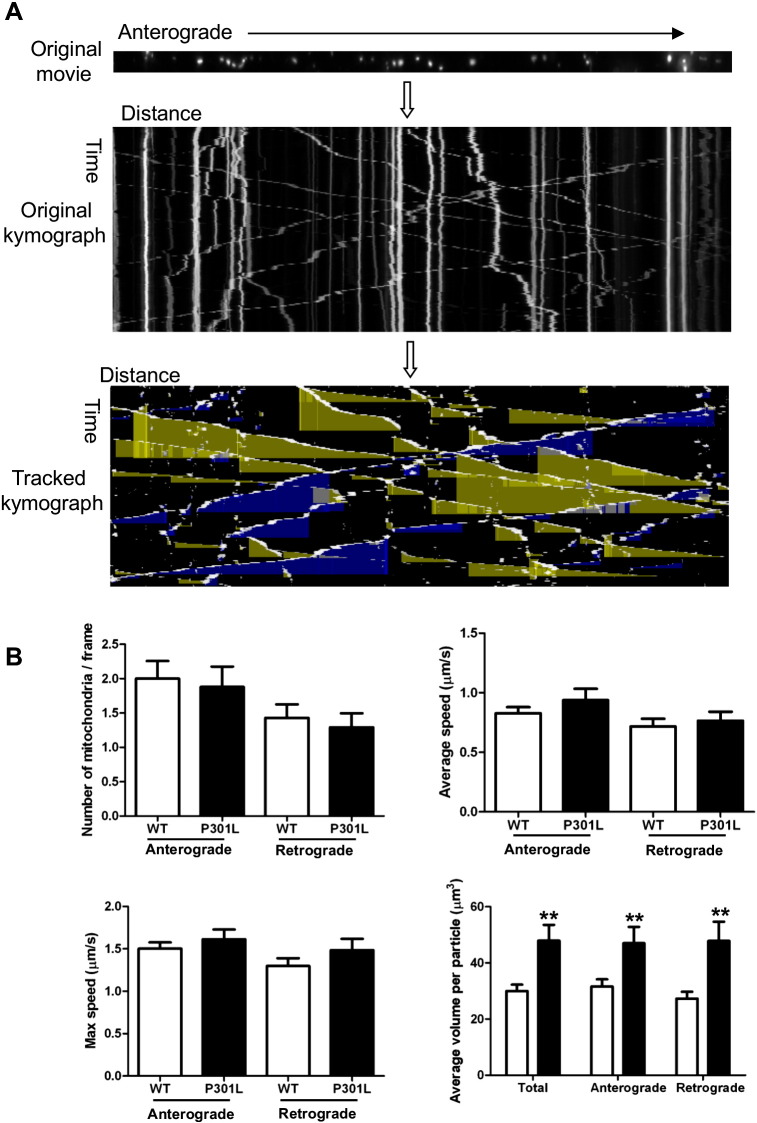
Mitochondrial movement kinetics are not affected in P301L tau axons. (A) Overview of the methodology used for mitochondrial transport analysis in mouse cortical neurons. Image stacks of individual axons were generated using the “Straighten” ImageJ plugin. Motile mitochondria were tracked using Difference Tracker plugins for ImageJ, showing anterograde transport (yellow shadow) and retrograde transport (blue shadow). (B) Quantification of the number, average speed, maximum speed and average volume per particle of motile mitochondria in wild-type (WT) and P301L tau-expressing neurons. Data are shown as mean ± SEM (n = 28 for wild-type, n = 15 for P301L, Student t-test, P < 0.01**).

We next characterised the transport parameters of motile mitochondria by analysing time-lapse images using the Difference Tracker programme. This programme provides an automated analysis of axonal transport parameters including average and maximum speeds and volumes of motile, but not stationary, mitochondria travelling in both anterograde and retrograde directions (Fig. 2A). The number of mitochondria moving in each direction was approximately equal in wild-type and KI-P301L tau neurons (Fig. 2B). The average and maximal speeds of motile mitochondria moving in each direction were also similar in wild-type tau and P301L tau-expressing neurons (Fig. 2B). However, the volume of individual mitochondria moving in both anterograde and retrograde directions was significantly increased in neurons expressing P301L tau (Fig. 2B), which could be related to a defect in mitochondrial fusion or fission. Lysates of 8 DIV wild-type and P301L neurons were analysed on Western blots probed with an antibody recognising optic atrophy 1 (Opa1) and mitofusin 2 (Mfn2), which are required for mitochondrial fusion (Fig. S1). No differences were detected in the amount of Opa1 and Mfn2 present in wild-type and P301L tau-expressing neurons. This result suggests that changes in mitochondrial fusion are unlikely to be responsible for the observed increase in mitochondrial volume due to the P301L tau mutation. However, it is worth noting that because only 20% of the mitochondria were motile in these neurons, possible changes in Opa1 and Mfn2 may not have been detectable using this approach.

Taken together, our quantitative analysis of total mitochondria and moving mitochondria in cultured neurons suggests that the P301L tau mutation causes a dramatic reduction in the overall number of axonal mitochondria. P301L tau also increases the volume of individual motile mitochondria, without affecting their speed, likelihood of movement or direction of movement.

### P301L tau is hypophosphorylated in cortical neurons

**Fig. 3:**
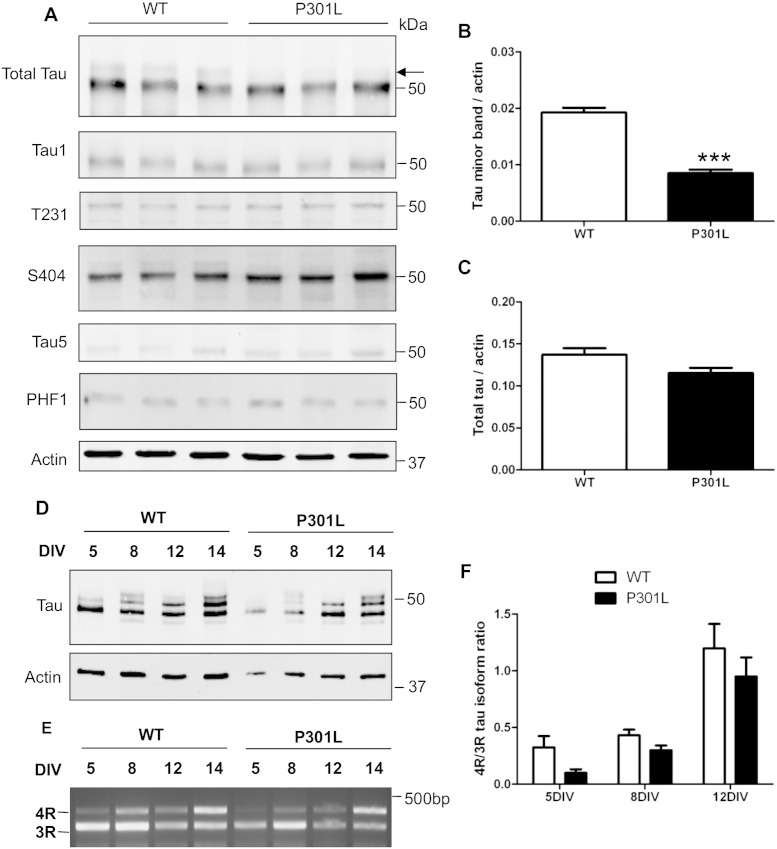
P301L tau is hypophosphorylated in mouse cortical neurons. (A) Lysates of wild-type (WT) and knock-in P301L (KI-P301L) tau neurons at 8 days in vitro (DIV) were analysed on Western blots probed with antibodies against total tau (DAKO and Tau5), dephosphorylated tau (Tau1), phosphorylated tau (pT231, pS404 and PHF1) and β-actin. The minor band of tau (arrow) was observed in WT, but not KI-P301L tau neurons. Quantification of the minor tau band (B) and total tau relative to β-actin (C). (D) Tau profile in WT and KI-P301L tau neurons after lambda phosphatase treatment at 5, 8, 12 and 14 DIV analysed on Western blots using an antibody to total tau (DAKO) and β-actin. (E) RT-PCR showing the relative expression of three-repeat (3R) and four-repeat (4R) tau in WT and P301L tau neurons at 5, 8, 12 and 14 DIV. (F) Quantification of the 4R/3R tau ratio determined by RT-PCR in WT and P301L tau neurons. Mean ± SEM (n = 3–6, Student t-test, P < 0.001***).

Our previous study of adult KI-P301L mice reported changes in axonal mitochondrial transport and a reduction in tau phosphorylation in adult mouse brain. To examine tau phosphorylation status in the cortical neurons used for the axonal transport studies, 8 DIV wild-type and KI-P301L tau neuronal lysates were probed with antibodies to tau and β-actin on Western blots (Fig. 3A). The tau antibodies recognise total tau (DAKO, Tau5), dephosphorylated tau (Tau1 [S199/S202]), or phosphorylated tau (pT231, pS404, PHF1 [S396/404]). The amount of tau was quantified and expressed relative to β-actin, whereas phospho-tau was standardised to total tau.

**Fig. S2:**
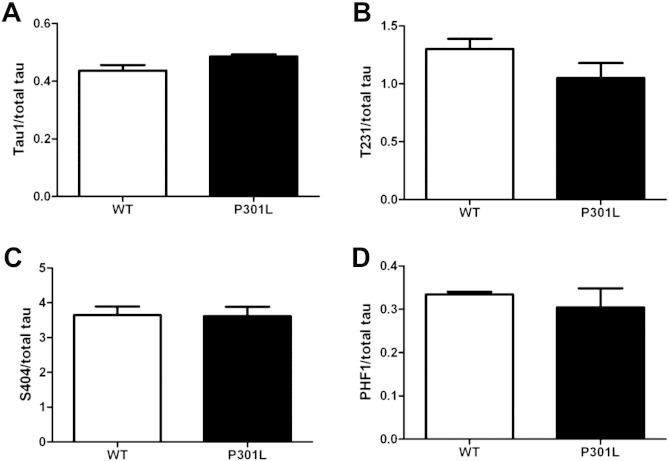
Analysis of tau phosphoepitopes in wild-type and P301L neurons. Quantification of western blots of (A) dephosphorylated tau (Tau1), and phosphorylated tau (B) pT231, (C) pS404, and (D) PHF1, relative to total tau in lysates of 8 days in vitro (DIV) wild-type (WT) and knock-in P301L (KI-P301L) tau neurons (mean ± SEM, n = 4).

Tau in wild-type neurons appears as a major species of ~ 50 kDa along with a minor, slower-migrating band (Fig. 3A, arrow). Quantification of the minor tau species revealed a significant decrease in P301L tau compared to wild-type neurons (Fig. 3B). However, the total amount of tau at 8 DIV was similar in both wild-type and P301L tau-expressing neurons (Fig. 3C). Quantification of tau phosphorylation did not reveal any statistically significant changes at the epitopes recognised by Tau1, pT231, pS404 or PHF1 antibodies in wild-type or P301L tau-expressing neurons (Fig. S2). Taken together, these results show that wild-type tau is phosphorylated at residues 199/202, 231, 396/404. Thus, these residues are not affected by the presence of P301L tau, despite KI-P301L tau neurons exhibiting a reduction in the amount of the slower migrating band, suggesting that the reduced tau phosphorylation induced by P301L tau does not result from changes at the epitopes examined here.

In embryonic day 18 (E18) brain, tau consists primarily of the smallest tau isoform, 0N3R. Other tau isoforms comprising both 3R and 4R species become apparent only at later stages of development (Hernandez et al., 2004). Since alternative splicing of 3R and 4R tau isoforms could contribute to the altered band pattern and electrophoretic migration of P301L tau, we investigated whether P301L tau affects alternative splicing of tau in cultured neurons. The profile of tau isoforms in wild-type and P301L tau-expressing neurons at 5, 8, 12 and 14 DIV was analysed on Western blots following neuronal lysis and dephosphorylation (Hanger et al., 2002). The results show that there is no apparent difference in the pattern of dephosphorylated tau bands in neurons from wild-type or KI-P301L tau mice (Fig. 3D). Furthermore, longer culture duration results in the appearance of larger tau isoforms with reduced mobility (Fig. 3D), in line with the expected increased production of 4R tau isoforms following extended culture (Deshpande et al., 2008). The observed pattern of tau species was consistent between wild-type and P301L tau-expressing neurons indicating that, at least up to 14 DIV, the P301L mutation does not affect alternative splicing of tau in cultured neurons.

To confirm and extend these observations, we extracted RNA from wild-type and P301L tau-expressing neurons at 5, 8, 12, and 14 DIV. Tau cDNA was analysed using primers spanning exons 9 to 13 to differentiate between alternatively spliced tau species that either lacked (3R) or included (4R) exon 10. The intensity of the PCR products corresponding to 3R and 4R tau isoforms was quantified after separation on agarose gels (Fig. 3E, F). Notably, the 4R/3R tau ratio remained below 0.5 up to 8 DIV, indicating a predominance of 3R tau. However, by 12 DIV, the 4R/3R tau ratio increased, showing that approximately equal amounts of 4R and 3R tau are expressed at this stage. The data suggest that a switch occurs between 8 and 12 DIV that drives the production of 4R isoforms in cultured primary neurons. These results thus confirm our findings that wild-type and P301L tau-expressing neurons exhibit similar 4R/3R isoform ratios, and show that the P301L mutation does not affect the normal splicing of tau.

**Fig. S3:**
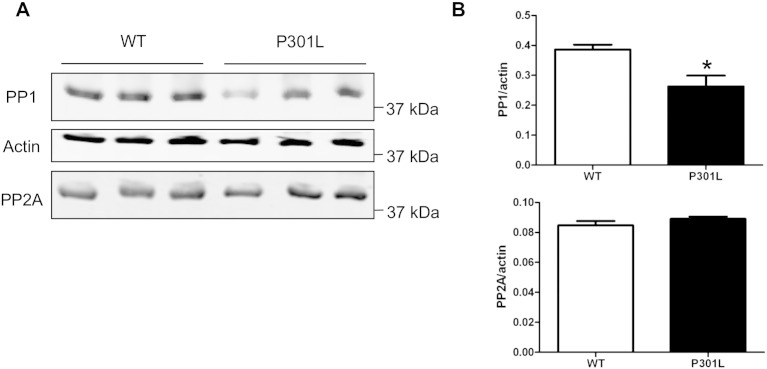
Reduced phosphorylation of P301L tau is not due to increased expression of protein phosphatases PP1 or PP2A. (A) Lysates of wild-type (WT) and knock-in P301L (KI-P301L) tau from 8 days in vitro (DIV) neurons were analysed on western blots probed with antibodies recognising PP1, PP2A, and β-actin. (B) Quantification of PP1 and PP2A normalised to β-actin in WT, and KI-P301L tau neurons (mean ± SEM, n = 3). Student t-test, P < 0.05*.

To further investigate the cause of the reduced phosphorylation of tau, protein phosphatase (PP) 1 and PP2A were analysed, since these have both been shown to dephosphorylate tau (Goedert et al., 1995, Gong et al., 1994, Liu et al., 2005). We quantified PP1 and PP2A in wild-type and P301L tau-expressing neurons relative to β-actin (Fig. S3). This analysis showed that the amount of PP1 was decreased by 32% in neurons expressing P301L tau, whereas the amount of PP2 was unchanged, relative to wild-type neurons.

### P301L tau and microtubule binding in cortical neurons

**Fig. 4:**
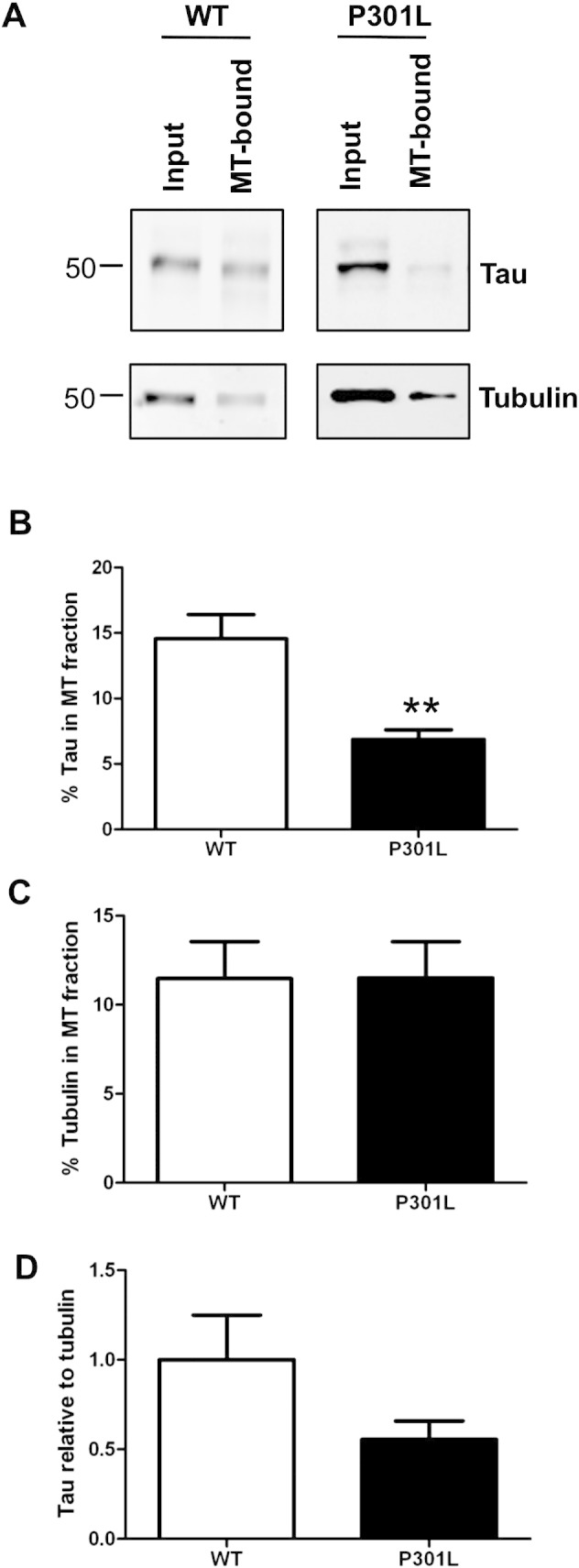
Microtubule-binding properties of wild-type and P301L tau in cortical neurons. (A) Wild-type (WT) and knock-in P301L (KI-P301L) tau neuronal lysates were separated into microtubule-bound and unbound fractions, and analysed on Western blots using antibodies to total tau and tubulin. Quantification of the percentage of total tau (B) or tubulin (C) present in the microtubule (MT) fraction. (D) Quantification of microtubule-bound tau relative to tubulin, expressed relative to WT tau. Data are shown as mean ± SEM (n = 7–8, Student t-test, P < 0.01**).

The microtubule-binding repeat domain of tau is important for its association with tubulin and 4R tau has been reported to bind more tightly to microtubules than 3R tau (Butner and Kirschner, 1991). Since the P301L tau mutation is located in exon 10, which encodes the fourth microtubule-binding repeat, it is possible that the P301L mutation could affect interactions between tau and microtubules. To address this question, lysates of wild-type and P301L tau cortical neurons (12 DIV) were fractionated into microtubule-bound, and unbound components, based on their differential solubility (Ding et al., 2006) and analysed on Western blots probed with antibodies for tau and tubulin (Fig. 4A). The percentage of tau and tubulin in the microtubule fractions relative to the input was quantified for each condition (Fig. 4B, C). The results show a statistically significant decrease in the amount of tau present in the microtubule fraction of KI-P301L tau neurons (14.6% for WT, 6.9% for P301L, P < 0.01) (Fig. 4B). This finding is in agreement with previous in vitro studies describing reduced microtubule binding due to the presence of the P301L tau mutation (D'Souza et al., 1999, Hong et al., 1998). No difference was found in the amount of tubulin present in the microtubule fraction (Fig. 4C). The results thus confirm that the P301L mutation reduces the capacity of tau to bind to microtubules under physiological conditions.

### P301L tau and membrane localisation

**Fig. 5:**
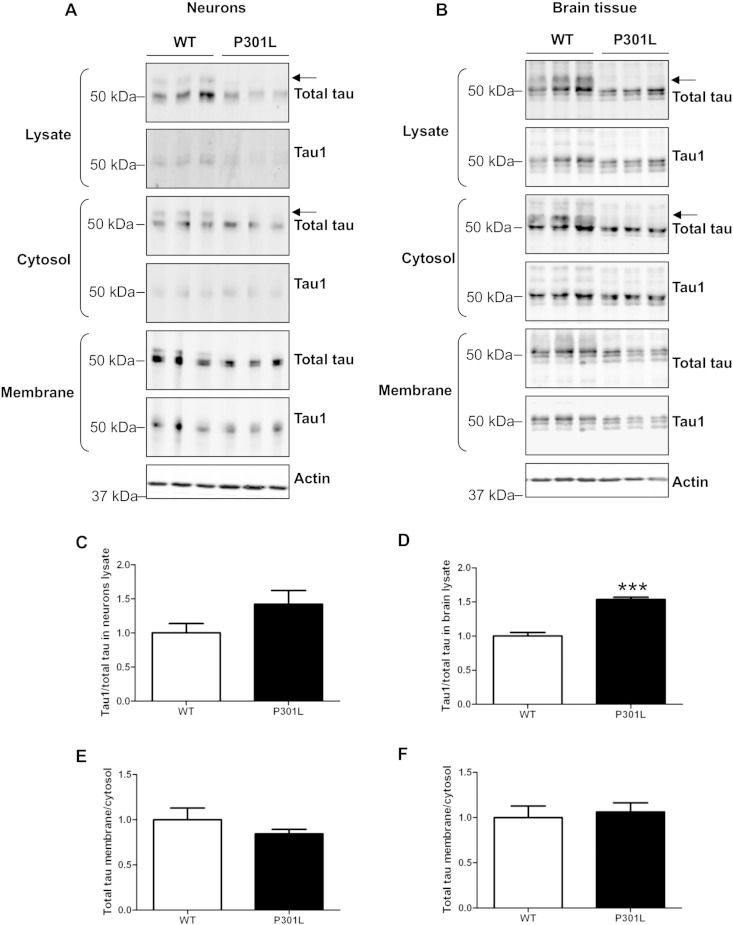
The P301L mutation in tau does not affect its membrane localization. Lysate, cytosol and membrane fractions of (A) 14 days in vitro (DIV) mouse cortical neurons and (B) adult mouse brain (4 months old), were analysed on Western blots probed with antibodies against total tau, dephosphorylated tau (Tau1) and β-actin. Quantification of dephosphorylated tau relative to total tau in lysates of neurons (C) and brain tissue (D). The ratio of the amount of tau localised in the membrane and the cytosol was calculated for wild-type (WT) and knock-in P301L (KI-P301L) tau-expressing neurons (E), and brain tissue (F). Data are shown as mean ± SEM (n = 11 for neurons, n = 6 for brain tissue, Student t-test, P < 0.001***).

We have previously reported that phosphorylation of tau affects its intracellular localization and that decreased tau phosphorylation increases its association with membranes (Pooler et al., 2012). Since KI-P301L-derived tau is relatively hypophosphorylated we determined if the presence of the P301L mutation could affect tau trafficking to the membrane in neurons. Lysates of wild-type and KI-P301L tau cortical neurons (14 DIV), and brain tissue from mice of four months of age, were separated into cytosolic and membrane fractions and analysed on Western blots probed with antibodies to total tau (DAKO) and dephosphorylated tau (Tau1) (Fig. 5A, B). The neurons from wild-type and KI-P301L tau neurons at 14 DIV contained tau species resembling those in 8 DIV neuronal cultures (Fig. 3A), in which KI-P301L tau neurons lack the slower migrating tau species (Fig. 5A, arrow). KI-P301L tau mouse brain also lacked this tau species and migrated slightly faster than wild-type tau (Fig. 5B, arrow). Quantification of dephosphorylated (Tau1), relative to total tau, revealed a significant increase in four month old KI-P301L tau brain, indicating increased tau dephosphorylation (Fig. 5D), but there was no statistically significant change in Tau1 in 14 DIV neurons (Fig. 5C). These results suggest that changes in tau phosphorylation are age-dependent and could be related to the increasing amount of 4R tau expressed in adult mice. The proportion of P301L tau in the membrane fraction was similar to that of wild-type tau in both neurons and adult mouse brain (Fig. 5D, F) suggesting that the hypophosphorylated state of P301L tau does not affect its trafficking to the membrane.

## Discussion

In a previous study of adult KI-P301L mice, which express the tau mutation at physiological levels, we reported a reduction in tau phosphorylation in adult mouse brain and age-dependent changes in axonal mitochondrial transport in isolated tibial nerve (Gilley et al., 2012). Here, we have investigated the effects of the P301L tau mutation in mouse cortical neurons from KI-P301L mice. Notably, we found that P301L tau expression reduces the number of axonal mitochondria, although their anterograde and retrograde directional kinetic parameters were similar to wild-type neurons. Approximately 20% of mitochondria were motile, irrespective of whether wild-type or mutant tau was expressed, similar to other reports of mitochondrial transport in sciatic nerve (Magrane et al., 2014), hippocampal neurons (Shao et al., 2013) and dopaminergic neurons (Kim-Han et al., 2011). However, the motile mitochondria in neurons harbouring P301L tau had an increased volume. It is conceivable that the larger volume of individual moving mitochondria is masked when measuring the size of all mitochondria (i.e., both motile and stationary) due to the large proportion of these stationary organelles. This increase in mitochondrial volume in P301L tau-expressing neurons is in agreement with a previous study of mice overexpressing the P301S tau mutation, in which the number of mitochondria in spinal cord motor neurons of adult P301S tau mice were reduced and also appeared to be swollen (Yoshiyama et al., 2007). However, contrasting results have also been reported with no change in the number of mitochondria in P301L tau over-expressing mice at 18 months of age (David et al., 2005). Interestingly, the angle which defines the orientation of the mitochondria in the axon was greater in neurons expressing KI-P301L tau. Such a change in orientation could lead to a temporary blockage or reduced flow of axonal mitochondria that, even if not affecting their overall transport kinetics, could increase the probability of mitochondrial fusion and decrease the number of mitochondria in the axon.

The effect of tau on mitochondrial function and dynamics has also been studied in neuroblastoma cells over-expressing wild-type and P301L tau (Schulz et al., 2012). These authors reported that the P301L tau mutation results in a deficit in mitochondrial complex 1, accompanied by reduced ATP levels, and increased susceptibility to oxidative stress. Furthermore, overexpression of P301L tau in neuroblastoma cells resulted in perinuclear clustering of mitochondria and reduced mitochondrial movement (Schulz et al., 2012). P301L tau has also been shown to decrease the rates of both fission and fusion of mitochondria, an observation corroborated by parallel reductions in the amounts of mRNA encoding proteins involved in these processes in neuroblastoma cells (Schulz et al., 2012). Taken together, these results suggest that P301L tau may have a fundamental effect on axonal mitochondria.

We found that P301L tau expression results in the appearance of hypophosphorylated tau in cultured cortical neurons, similar to our findings in adult mouse brain (Gilley et al., 2012). This suggests that the observed decrease in tau phosphorylation in the KI-P301L tau mice is an early event that is maintained into adulthood. To investigate the cause of the reduced phosphorylation of tau, two enzymes known to dephosphorylate tau, PP1 and PP2A, were examined. We found that expression of these phosphatases was not increased in KI-P301L mice. Instead we observed a reduction of 32% in the amount of PP1, and no change in PP2A, suggesting that hypophosphorylation of tau in KI-P301L mice is not due primarily to increased expression of these phosphatases. It is conceivable that decreased tau phosphorylation could be the consequence of reductions in the amounts or activities of tau kinases or, alternatively, an altered tau conformation that prevents its physiological phosphorylation. For example, reduced PP1C activity increases serine 9 phosphorylation of glycogen synthase kinase-3 beta (GSK-3), which reduces GSK-3 activity (Manser et al., 2012). Since GSK-3 is a tau kinase (Hanger et al., 1992), reduced PP1C could result in decreased tau phosphorylation.

We also analysed the possible effect of the P301L mutation on tau alternative splicing by determining the 4R/3R tau ratio in neurons at different ages in vitro. We confirmed here that physiological expression of P301L does not interfere with normal tau splicing in cortical neurons, a finding in line with previous in vitro (D'Souza et al., 1999) and in vivo (Hutton et al., 1998) studies.

The effect of the P301L mutation on tau-microtubule binding properties of tau was determined in cortical neurons. Our previous comparison of wild-type and KI-P301L tau in adult mouse brain showed a decreased amount of P301L tau in microtubule-containing fractions (Gilley et al., 2012). This is recapitulated here in 12 DIV cortical neurons derived from embryonic brain tissue in which the amount of tau bound to microtubules was significantly reduced in KI-P301L tau neurons, whereas the amount of tubulin was unaffected. These results are in agreement with previous reports suggesting that P301L tau has a reduced ability to bind to microtubules (D'Souza et al., 1999, Dayanandan et al., 1999, Hong et al., 1998). The tau/tubulin ratio was diminished in KI-P301L neurons but this difference did not reach significance. The reason for this could be explained by the fact that 12 DIV cortical neurons express approximately 50% of tau as 3R isoforms, which lack the part of the microtubule-binding domain encoded by exon 10. In contrast, mouse tau in adult KI-P301L brain is comprised of 4R tau isoforms, which includes the affected residue, and shows a significant decrease in the tau/tubulin ratio (Gilley et al., 2012).

Phosphorylation of tau reduces its association with the neuronal plasma membrane (Pooler et al., 2012). Since P301L tau appears to be less phosphorylated in adult KI-P301L mice and also in cortical neurons derived from these animals, we investigated whether the P301L mutation affected the intracellular localization of tau. We found that the relative proportion of tau in membrane and cytosolic fractions of both cultured neurons and adult mouse brain was similar for wild-type and P301L tau. This shows that tau hypophosphorylation caused by the presence of the mutation does not influence its intracellular localisation. However, phosphorylation in the N-terminal half of tau has been shown to influence its ability to localise to membranes (Pooler et al., 2012) and therefore, since P301L is in the C-terminal region of tau, it is possible that the phosphorylation sites affected by the presence of this mutation may not have a significant influence on the ability of tau to associate with neuronal membranes. Interestingly, the increase in dephosphorylated tau was observed only in 4 month old KI-P301L brains, and not in cultured cortical neurons. This suggests an age-dependent increase in dephosphorylated tau, possibly related to an increase in 4R tau isoforms containing the P301L mutation in adult mice.

## Conclusions

Expression of endogenous levels of P301L tau in mouse cortical neurons reduces tau phosphorylation but does not influence the association of tau with membranes or tau splicing. P301L tau expression results in reduced numbers of axonal mitochondria and increased volume of individual motile mitochondria, without affecting their kinetic parameters. Our results imply that some of the effects of P301L tau on mitochondria reported in previous studies could be due to over-expression of mutant tau. Significantly, physiological expression levels of P301L tau result in relatively modest changes to mitochondria and tau phosphorylation state in both cultured neurons and in adult mice. Understanding the mechanisms that lead to reduced numbers and larger volumes of motile mitochondria due to the presence of P301L tau warrants further investigation since disruption of mitochondrial function is likely to be severely detrimental to neuronal survival.

The following are the supplementary data related to this article.

## Figures and Tables

**Table 1:** Motility of mitochondria in neurons expressing wild-type and KI-P301L tau.

Neuronal genotype	% Anterograde	% Retrograde	% Stationary
Wild-type tau	11.2 ± 1.5 (28)	8.0 ± 1.1 (28)	80.7 ± 1.7 (28)
KI-P301L tau	12.5 ± 2.5 (15)	9.6 ± 2.1 (15)	77.9 ± 3.0 (15)

Time-lapse recordings of mitochondrial transport in wild-type and knock-in P301L (KI-P301L) tau neurons were tracked. The percentage of motile mitochondrial travelling in each direction, and percentage of stationary mitochondria are shown. Data are expressed as mean ± SEM (n).
